# Making cities mental health friendly for adolescents and young adults

**DOI:** 10.1038/s41586-023-07005-4

**Published:** 2024-02-21

**Authors:** Pamela Y. Collins, Moitreyee Sinha, Tessa Concepcion, George Patton, Thaisa Way, Layla McCay, Augustina Mensa-Kwao, Helen Herrman, Evelyne de Leeuw, Nalini Anand, Lukoye Atwoli, Nicole Bardikoff, Chantelle Booysen, Inés Bustamante, Yajun Chen, Kelly Davis, Tarun Dua, Nathaniel Foote, Matthew Hughsam, Damian Juma, Shisir Khanal, Manasi Kumar, Bina Lefkowitz, Peter McDermott, Modhurima Moitra, Yvonne Ochieng, Olayinka Omigbodun, Emily Queen, Jürgen Unützer, José Miguel Uribe-Restrepo, Miranda Wolpert, Lian Zeitz

**Affiliations:** 1 Department of Mental Health, Johns Hopkins Bloomberg School of Public Health, Baltimore, MD USA; 2 citiesRISE, New York, NY USA; 3 Department of Global Health, University of Washington, Seattle, WA USA; 4 Centre for Adolescent Health, University of Melbourne, Melbourne, Victoria Australia; 5 Dumbarton Oaks, Harvard University, Washington, DC USA; 6 Centre for Urban Design and Mental Health, London, UK; 7 Orygen, Melbourne, Victoria Australia; 8 University of Melbourne, Melbourne, Victoria Australia; 9 Ecole de Sante Publique, Universite de Montreal, Montreal, Quebec Canada; 10 Fogarty International Center, National Institutes of Health, Bethesda, MD USA; 11 Aga Khan University, Nairobi, Kenya; 12 Grand Challenges Canada, Toronto, Ontario Canada; 13 Good South Social Impact Enterprise, Durban, South Africa; 14 Universidad Peruana Cayetano Heredia, Lima, Peru; 15 Sun Yat Sen University, Guangzhou, China; 16 Mental Health America, New York, NY USA; 17 World Health Organization, Geneva, Switzerland; 18 The TruePoint Center, Boston, MA USA; 19 Healthy Brains Global Initiative, Nairobi, Kenya; 20 Teach for Nepal, Kathmandu, Nepal; 21 Department of Population Health, New York University Grossman School of Medicine, New York, NY USA; 22 University of Nairobi, Nairobi, Kenya; 23 Sacramento County Board of Education, Sacramento, CA USA; 24 Lefkowitz Consulting, Sacramento, CA USA; 25 Fajara Associates, London, UK; 26 Duke University, Durham, NC USA; 27 University of Ibadan, Ibadan, Nigeria; 28 Pontificia Universidad Javeriana, Bogotá, Colombia; 29 Wellcome Trust, London, UK; 30 Climate Mental Health Network, Annapolis, MD USA

**Keywords:** Risk factors, Psychology, Public health

## Abstract

Urban life shapes the mental health of city dwellers, and although cities provide access to health, education and economic gain, urban environments are often detrimental to mental health^1,2^. Increasing urbanization over the next three decades will be accompanied by a growing population of children and adolescents living in cities^3^. Shaping the aspects of urban life that influence youth mental health could have an enormous impact on adolescent well-being and adult trajectories^4^. We invited a multidisciplinary, global group of researchers, practitioners, advocates and young people to complete sequential surveys to identify and prioritize the characteristics of a mental health-friendly city for young people. Here we show a set of ranked characteristic statements, grouped by personal, interpersonal, community, organizational, policy and environmental domains of intervention. Life skills for personal development, valuing and accepting young people’s ideas and choices, providing safe public space for social connection, employment and job security, centring youth input in urban planning and design, and addressing adverse social determinants were priorities by domain. We report the adversities that COVID-19 generated and link relevant actions to these data. Our findings highlight the need for intersectoral, multilevel intervention and for inclusive, equitable, participatory design of cities that support youth mental health.

## Abstract (web summary)

A study examining how cities can foster well-being and positive mental health in young residents synthesizes opinions from researchers, practitioners, advocates and young people, highlighting factors that policymakers and urban planners should consider.

## Main

More than a decade ago, Galea posed the question “Can we improve mental health if we improve cities?”^4^. In the past two centuries, urbanization has shaped landscapes and lives, making it the “sentinel demographic shift” of our times^4^. The relationships between mental health status and the social, cultural and physical environment have been explored for at least as long; nineteenth-century researchers proposed environmental exposures as possible explanations of ‘insanity’^5^. Faris and Dunham’s classic 1930s study^6^ linked social disorganization and unstable communities to mental disorders. Two decades later, Leonard Duhl sought to create healthy societies through liveable cities, informing the World Health Organization’s Healthy Cities initiative^7,8^. The question remains pertinent today even as we recognize the multiple and complex forces that shape mental health^9^. Today we understand that urban environments influence a broad range of health outcomes for their populations, positively and negatively, and this impact is manifested unequally^10^. Opportunities for education and connection exist for some, whereas rising levels of urban inequality, violence, stressful racial or ethnic dynamics in urban neighbourhoods, exposure to environmental toxins, lack of green space, inadequate infrastructure and fear of displacement increase risk for poor mental health and disproportionately affect marginalized groups^11^. Disparate outcomes also pertain to distinct developmental stages, and the mental health of adolescents and young adults is particularly vulnerable to urban exposures.

## Adolescents, youth and urban mental health

Young people under the age of 25 are the demographic group most likely to move to cities for educational and employment opportunities, and by 2050 cities will be home to 70% of the world’s children^3^. Cities concentrate innovation^3^ and have long been considered the consummate source of skills, resources and talent^12^. They offer greater opportunities for health and economic development, education, employment, entertainment and social freedoms (that is, the ‘urban advantage’), but rapid urbanization also deepens disparities and exposes individuals to considerable adversity, placing their mental health at risk^13^. In fact, most evidence points to urban living as a risk factor for poorer mental health, yielding increased risk for psychosis, anxiety disorders and depression^1,2^. Adolescence and young adulthood, specifically, encompass a critical period of risk for the incidence of mental disorders: an estimated half of mental disorders evident before age 65 begin in adolescence and 75% begin by age 24 (ref. ^14^). Mental disorders are the leading causes of disease burden among 10–24-year-olds worldwide^15^, responsible for an estimated 28.2 million disability-adjusted life years globally, with 1 disability-adjusted life year being equivalent to a healthy year of life lost to the disability caused by mental disorders. Public awareness of these issues rose as the incidence of mental disorders and suicide increased in some countries among adolescents and young adults during the coronavirus pandemic^16,17^. Urban environments probably have a role in these processes.

Fundamental to adolescents’ growth and development are their interactions with the complex urban environment: physical, political, economic, social and cultural^18^. Adolescents have a heightened sensitivity to context and social evaluation, and a stronger neural response to social exclusion, as well as to threat and reward stimuli^19^, and it is plausible that they may be particularly sensitive to social and environmental cues in the urban context, such as discrimination or violence. Discriminatory policies and norms are entrenched in many of the institutions with which young people interact (for example, schools, housing, justice and policing), and minoritized youth may experience the emotional and mental health consequences^20^. In fact, in settings of structural inequality (for example, high neighbourhood poverty and unemployment), young people are at greater risk for low self-efficacy and feelings of powerlessness and depression^21^. Social cohesion and collective efficacy can reduce the effects of concentrated disadvantage and nurture social and emotional assets among young people, families and their networks^21^.

At present, the world’s largest population of adolescents and young adults so far is growing up amid the sequelae of a tenacious pandemic, rapid population growth in urban centres and increasing urbanization, demanding an urgent response to support youth mental health^22^. Investing in adolescent well-being is said to yield a triple dividend through actions that reduce mortality and disability in adolescence, prolong healthy life in adulthood, and protect the health of the next generation by educating and strengthening the health of young parents^23^. Interventions in urban settings that align with developmental needs of adolescents and young adults could remediate insults from early life and establish healthy behaviours and trajectories for adult life^19,24^, potentially averting chronic conditions such as human immunodeficiency virus (HIV) and the associated mental health, social and physical sequelae^25^. In fact, investment in a package of adolescent mental health interventions can yield a 24-fold return in health and economic benefits^26^. At the societal level, shaping the aspects of urban life that influence youth mental health—through services, social policies and intentional design—could have an enormous impact^4^. Proposals for ‘restorative urbanism’ that centre mental health, wellness and quality of life in urban design may move cities in the direction of moulding urban environments for better adolescent health^27,28^. Young people, who contribute to the creativity of urban environments and drive movements for social change^29^, have a central part to play in this transformation.

Mental Health Friendly Cities, a global multi-stakeholder initiative led by citiesRISE, mobilizes youth-driven action and systems reform to promote and sustain the mental health and well-being of young people in cities around the world^30,31^ (Supplementary Information). To guide transformative actions that will enable cities to promote and sustain adolescent and youth mental health, we studied global priorities for urban adolescent mental health. One aim of this study is to contribute data-driven insights that can be used to unite several sectors in cities to act within and across their domains in favour of mental health promotion and care that is responsive to the needs of young people. To that end, we administered a series of linked surveys that permitted the influence of ideas from young people and multidisciplinary domain experts through an anonymous sequential process, following established methods for research priority setting^32^.

## Framework and top-ranked recommendations

**Fig. 1:**
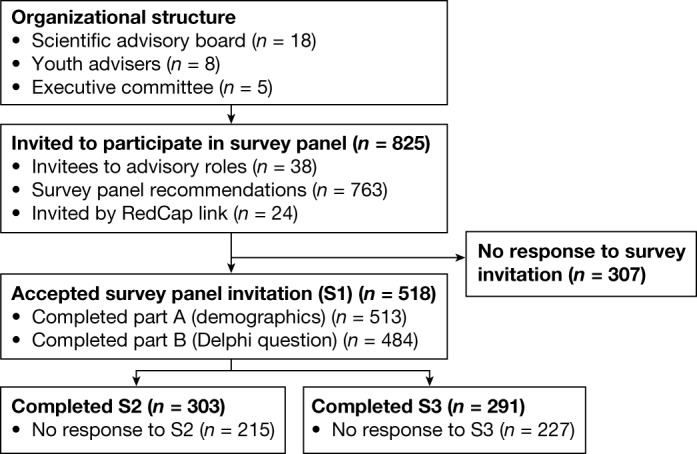
Project leadership and recruitment procedures. The composition of the project leadership structures, sample recruitment and participation by each survey round are shown below. We invited 801 individuals to participate in the survey panel through recommendations and direct invitations from advisory board members. Participants recruited through snowball sampling received the Research Electronic Data Capture (REDCap) link (*n* = 24). Individuals who gave informed consent in REDCap were deemed to have accepted the survey panel invitation. S1, survey 1; S2, survey 2; S3, survey 3.

**Fig. 2:**
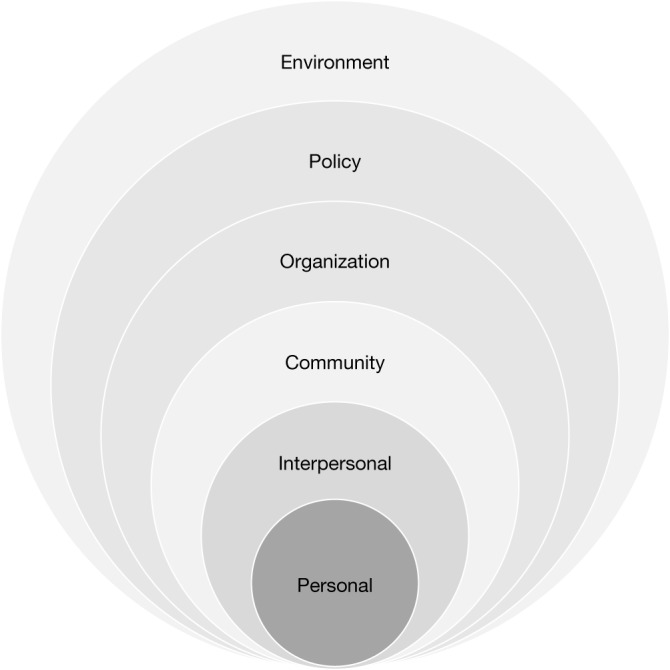
Socioecological model. The socioecological model with six levels (personal, interpersonal, community, organization, policy and environment) that are used to categorize the characteristics of a mental health friendly city.

To determine the elements of an urban landscape that would support mental health for adolescents and youth and would amplify their voices, we recruited a panel of 518 individuals from 53 countries to participate in a series of three digitally administered surveys that began in April 2020 (Table 1). Figure 1 shows the panel participation at each round. In survey 1, panellists responded to the open-ended question: “What are the characteristics of a mental health-friendly city for young people?”. Analysis of survey 1 data produced 134 statements about mental health-friendly cities for young people (Methods). In survey 2, participants selected their preferred 40 of the 134 statements. They were also presented with a second question related to the influence of the COVID-19 pandemic on their ideas about youth well-being in cities. In survey 3, we categorized survey 2 statements by socioecological domains (Fig. 2) and asked panellists to rank-list their preferred statements in each domain. Before ranking, panellists were required to choose one of three framings that informed their selected ranking: immediacy of impact on youth mental health; ability to help youth thrive in cities; and ease or feasibility of implementation.

**Table 1:** Participant demographics for survey panellists and retention data over three sequential surveys

	Overall *n* (%)	S1 *n* (%)	S2 *n* (%)	S3 *n* (%)
	518	484 (93.4%)	303 (58.5%)	291 (56.2%)
**Age**				
14–17 years	3 (0.6%)	3 (0.6%)	0 (0%)	0 (0%)
18–24 years	89 (17.2%)	82 (15.8%)	43 (8.3%)	42 (8.1%)
25–35 years	235 (45.4%)	221 (42.7%)	116 (22.4%)	107 (20.7%)
>35 years	186 (35.9%)	178 (34.4%)	143 (27.6%)	140 (27.0%)
Missing	5 (1.0%)	34 (6.6%)	216 (41.7%)	229 (44.2%)
**Gender**				
Female	285 (55.0%)	270 (52.1%)	175 (33.8%)	166 (32.0%)
Male	208 (40.2%)	196 (37.8%)	116 (22.4%)	112 (21.6%)
Non-binary	2 (0.4%)	2 (0.4%)	1 (0.2%)	1 (0.2%)
Missing	23 (4.4%)	50 (9.7%)	226 (43.6%)	239 (46.1%)
**Role**				
Educator	181 (34.9%)	169 (32.6%)	89 (17.2%)	81 (15.6%)
Researcher	150 (29.0%)	146 (28.2%)	111 (21.4%)	103 (19.9%)
Programme manager	103 (19.9%)	101 (19.5%)	66 (12.7%)	64 (12.4%)
Advocate	83 (16.0%)	81 (15.6%)	61 (11.8%)	58 (11.2%)
Student	79 (15.3%)	73 (14.1%)	43 (8.3%)	40 (7.7%)
Clinician	78 (15.1%)	73 (14.1%)	55 (10.6%)	53 (10.2%)
Other	66 (12.7%)	62 (12.0%)	37 (7.1%)	39 (7.5%)
Activist	44 (8.5%)	43 (8.3%)	27 (5.2%)	26 (5.0%)
Civil servant	17 (3.3%)	17 (3.3%)	11 (2.1%)	10 (1.9%)
Policymaker	15 (2.9%)	15 (2.9%)	11 (2.1%)	11 (2.1%)
Missing	0 (0%)	34 (6.6%)	215 (41.5%)	227 (43.8%)
**Domain**				
Education	186 (35.9%)	175 (33.8%)	87 (16.8%)	81 (15.6%)
Mental health or substance use	184 (35.5%)	178 (34.4%)	132 (25.5%)	131 (25.3%)
Student	97 (18.7%)	88 (17.0%)	57 (11.0%)	53 (10.2%)
Health care	87 (16.8%)	84 (16.2%)	64 (12.4%)	61 (11.8%)
Youth advocacy	79 (15.3%)	79 (15.3%)	47 (9.1%)	51 (9.8%)
Adolescent development	65 (12.5%)	65 (12.5%)	44 (8.5%)	43 (8.3%)
Other	55 (10.6%)	52 (10.0%)	40 (7.7%)	38 (7.3%)
Technology	29 (5.6%)	28 (5.4%)	17 (3.3%)	15 (2.9%)
Built environment	23 (4.4%)	21 (4.1%)	19 (3.7%)	16 (3.1%)
Not working at present	20 (3.9%)	19 (3.7%)	10 (1.9%)	9 (1.7%)
Urban planning	17 (3.3%)	16 (3.1%)	14 (2.7%)	12 (2.3%)
Urban development	15 (2.9%)	14 (2.7%)	11 (2.1%)	9 (1.7%)
Criminal justice	8 (1.5%)	7 (1.4%)	2 (0.4%)	2 (0.4%)
Big data	7 (1.4%)	7 (1.4%)	4 (0.8%)	3 (0.6%)
Housing	5 (1.0%)	4 (0.8%)	3 (0.6%)	3 (0.6%)
Missing	0 (0%)	34 (6.6%)	215 (41.5%)	227 (43.8%)

‘Overall’ indicates all panellists who accepted the invitation to participate in the survey. Gender is based on an open text response; most panellists responded with terminology typically designating biological sex. ‘Woman’ and ‘man’ are conventional gender terms. Role and domain are multiple selection parameters.

**Fig. 3:**
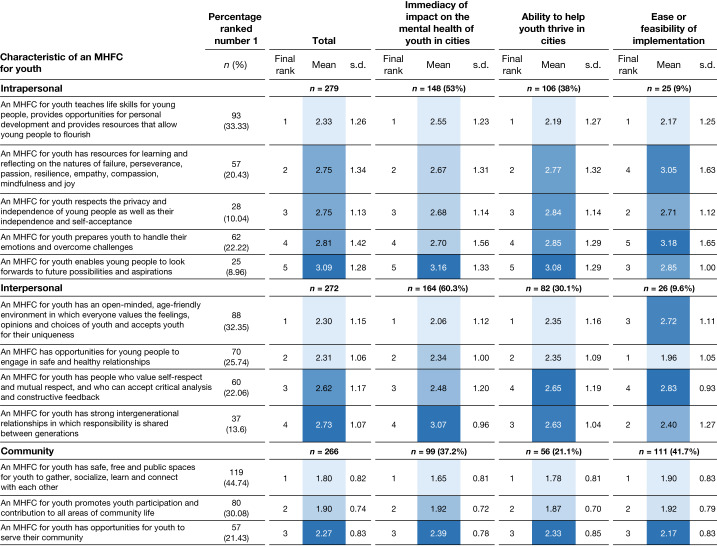
Characteristics of a mental health-friendly city rank-ordered by socioecological domain (intrapersonal, interpersonal and community) and grouped by three framing conditions. Mean ranks and standard deviations (s.d.) values for each mental health-friendly city (MHFC) characteristic are reported grouped by socioecological level and three framings described in the Analysis: immediacy of impact; ability to help youth thrive in cities; and ease or feasibility of implementation. Overall ranks (along with mean and s.d. values) for the total sample are reported. *n* values in bold represent the number of participants responding for each domain; the percentages in bold represent the percentage of respondents per domain. The number and percentage of the sample that assigned the highest rank for each characteristic are also reported (column 2). The colour continuum from light blue to dark blue shows the highest ranked means in the lightest shades and the lower ranks in darker blue.

**Fig. 4:**
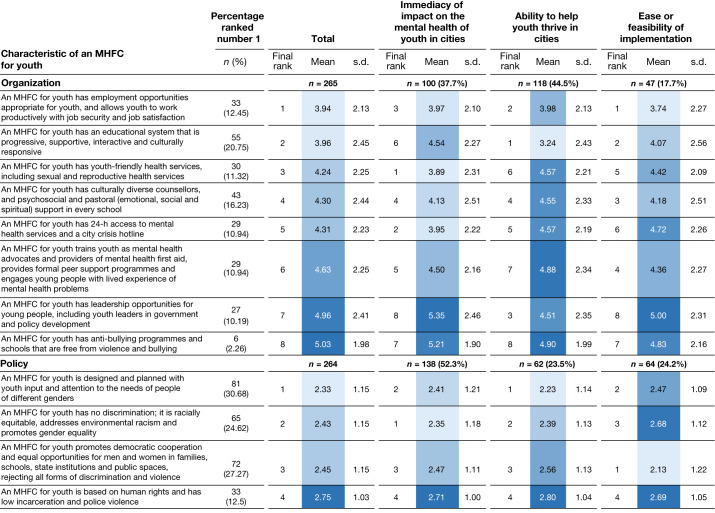
Characteristics of a mental health-friendly city rank-ordered by socioecological domain (organization and policy) and grouped by three framing conditions. See the caption of Fig. 3 for details.

**Fig. 5:**
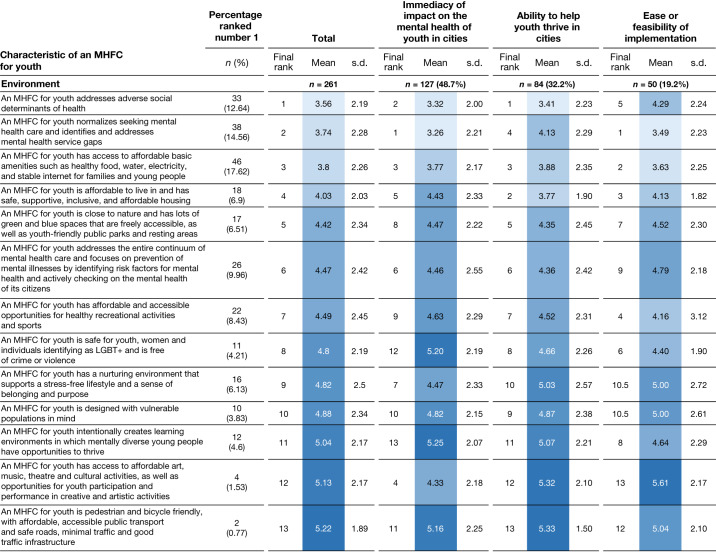
Characteristics of a mental health-friendly city rank-ordered by socioecological domain (environment) and grouped by three framing conditions. See the caption of Fig. 3 for details. LGBT+, people from sexual and gender minorities.

We present the findings of the third survey within a socioecological model (Figs. 3–5) because of this model’s relevance to the combination of social and environmental exposures in an urban setting and their interaction with the developing adolescent^33^. Bronfenbrenner’s model begins by recognizing that young people’s personal experiences and development are shaped by their interactions with the people around them^34^; that is, they react to and act on their immediate environment of familial and peer relationships (microlevel). These interpersonal relationships are also influenced by neighbourhood and community dynamics and exposure to institutions and policies (mesolevel). These, in turn, are nested within the organizational, political, historical, cultural (for example, values, norms and beliefs) and physical environments (macrolevel) whose interplay directly or indirectly affects the adolescent’s mental health and well-being. A high court ruling (policy environment) could have direct or indirect effects on the community, household and personal well-being of a young person seeking asylum. The socioecological framework encompasses the dynamic relationships of an individual with the social environment.

### The characteristics

We grouped 37 city characteristics across 6 socioecological domains: personal, interpersonal, community, organizational, policy and environmental. Figures 3–5 show the mean ranking for each framing and the total mean ranking averaged across frames. We show, for each characteristic statement, the number and percentage of panellists who ranked it highest. The five characteristics in the personal domain centre on factors that enable healthy emotional maturation for young people, future orientation and self-reflexivity. Most panellists (53%) ranked these characteristics according to immediacy of impact on youth mental health in cities, and mean rankings were identical to those linked to ability to help youth thrive in cities. The characteristic that describes prioritizing teaching life skills, providing opportunities for personal development and providing resources that allow young people to flourish rose to the top mean rank for each frame and was also ranked first in this domain by the largest number of panellists (*n* = 93). Notably, the characteristic that describes preparing youth to handle their emotions and overcome challenges was ranked first by 62 panellists, although its mean rank was much lower.

Characteristics in the interpersonal domain refer to young people’s interactions with others in the environment. Prioritized characteristics in this domain centred on relationships marked by acceptance and respect for young people and noted the value of intergenerational relationships. The top-ranked characteristic emphasized age friendliness and interactions that value the feelings and opinions of young people as well as safe and healthy relationships. In this domain, ranked means for characteristics framed according to immediacy of impact on youth mental health and ability to help youth thrive were the same for the top two characteristics. Notably, the two highest-ranked means for ease of implementation focused on opportunities for safe and healthy relationships and strengthening intergenerational relationships.

Young people’s intrapersonal experiences and interpersonal relationships are nested within a system of community and organizational relationships. Study participants prioritized access to safe spaces for youth to gather and connect among the three characteristics in the domain of community, and rankings were identical for each framing. At the organizational domain, two characteristics shared high mean rankings: employment opportunities that allow job security and satisfaction and a responsive and supportive educational system. Health-care services and educational services were the organizations most frequently referenced in relation to youth mental health. Whereas employment opportunities ranked first in terms of feasibility of implementation, provision of youth-friendly health services ranked first for immediacy of impact on youth mental health. With the exception of the community and organizational domains, more panellists chose to frame their responses in terms of immediacy of impact on youth mental health.

Of the four statements in the policy domain, the design and planning of cities with youth input and gender sensitivity ranked highest overall and was most frequently ranked first by panellists (30.68%). Promoting democratic cooperation and equal opportunity and anti-discrimination in all institutions received the highest mean rank for feasibility of implementation.

The sixth socioecological domain lists 13 characteristics related to the social, cultural and physical environments. Addressing adverse social determinants of health for young people had the highest overall ranked mean; however, normalizing youth seeking mental health care and addressing service gaps ranked first when framed by feasibility of implementation and immediacy of impact. Having access to affordable basic amenities was most frequently ranked first in this domain by panellists, but panellist preferences were distributed across the list.

## COVID-19 and urban youth well-being

Our data collection began in April 2020 during the COVID-19 pandemic, and by survey 2 (August 2020), most countries were experiencing the pandemic’s public health, social and economic effects. In light of this, we added an open-ended survey question to which 255 participants responded “How has the COVID-19 pandemic changed your ideas about the wellbeing of young people in cities?” (Methods). Most respondents reported changes in perspective or new emphases on inequities as determinants of youth well-being and mental health, whereas nine reported that COVID-19 did not change their ideas. For one such respondent (in the >35 years age category), the pandemic merely confirmed the powerful effect of social vulnerabilities on risk and outcomes during an emergency: “COVID-19 has not changed my ideas about the wellbeing of young people in cities. I found that the young people in cities who did well during the lockdown period and the difficult period of the pandemic were those who were already doing well in terms of a rich social network, good interpersonal relations with family and friends, enjoyable work life, a close religious network, membership [in] a young people’s club so that they were able to stay connected via social media. Those who had access to food and essential commodities and those who knew they would return to school or work after the pandemic. Those who had access to good living conditions and some space for recreation also did well. ... The impact of COVID19 was felt much more by those with existing mental health conditions, living in crowded slums, poverty, unemployment, who were uncertain about the next step”.

Respondents highlighted losses young people experienced as a result of the pandemic. These included loss of the city as a place of opportunity; loss of jobs, familial and individual income, and economic stability; loss of a planned future and loss of certainty; loss of rites of passage of youth; loss of access to friends, social networks and social support; loss of access to quality education and to health care, especially mental health care and sexual and reproductive health services; loss of opportunities for psychological and social development; and loss of loved ones who died from COVID-19. We summarize the qualitative findings according to the socioecological framework. We present sample quotes in Table 2, along with the age category of the respondents (18–24, 25–35 and >35) and actions for cities to take.

**Table 2:** Perceived risks to youth mental health during the COVID-19 pandemic and actions to support mental health

**Socioecological domain**	**Illustrative quotes**	**Actions to support mental health**
**Policy and environment**		
**Governance and equity**	“The pandemic has shone a brighter light on all the infrastructural and societal issues we have been generally aware of for a long time, but for which perhaps a renewed energy should be exerted … It showed that our education system is underprepared to serve diverse needs and is mostly geared to cater to those with the most privilege. It shows that our higher education system is highly funded by unpaid Black and brown young people performing on fields, courts and arenas for wealthy people’s entertainment. It reveals that society is willing to risk some lives called ‘essential workers’ who are mostly BIPOC and low income. Especially, it reveals that during times of high stress societally, decision makers seem to let go of issues related to equity.” (>35). “… One cannot separate the impact of the pandemic from the nationwide calls for justice and how the issues of racism further threaten our youth’s mental wellbeing. After reading all 134 characteristics below, I see there is no mention of a MHFC for youth having institutions and leaders who work to **dismantle the systems of oppression and racism** that cause ongoing and significant harm to our youth’s mental wellbeing.” (>35).	Dismantle systems of oppression and racism
**Social norms and protections**	“Implementation of initial lockdown measures forced youth along with children, adults and families to move from big cities to villages due to lack of minimum social welfare, employment and increasing hunger, homelessness etc. Labelled as ‘migrants’, cities were not able to guarantee and uphold basic rights for all youth, especially vulnerable groups. Many young people were forced to travel long distances on foot due to lack of transport or any other amenities.” (25–35).	
**Resourced built environment**	“I believe that young people and city officials have had a re-awakening when it comes to the need for open & public spaces in cities. There is certainly not enough open space in urban settings. **Young people need access to parks and open space**. These spaces provide opportunities for youth to connect with themselves as individuals, with other youth, and with the community.” (25–35). “The pandemic has **highlighted the need for more creative green spaces in cities along with more accessibility**. Closing parks at certain hours is a very uncomfortable public policy. Nature should not align with strict schedules designed by those who may not even use the spaces. Nature should have unlimited access.” (>35).	Policies to increase access to green and blue spaces
**Community and organizations**		
**Supportive educational and healthcare systems**	“There’s little or no access to mental health care for young people, even virtual services. Many young people lost loved ones to the virus at periods when hospitals and counseling centers were shut down. There were hardly any mass media messages providing psychological support for them or directing them to virtual access to psychological first-aid. There’s still low awareness of the scope of care and support they can get from mental health experts … Prevention activities are focused mainly on adults and the elderly, and children. Most messages created were hardly directed specifically at young people. Apparently, adolescents and young adults are still a ‘forgotten’ group of the society. Even if they were prone to the health issues or complications, they were **hardly targeted specifically with health-related messages**.” (>35). “I think **short courses on crisis intervention and adaptation techniques** could be implemented throughout the curriculum from as early as age twelve.” (18–24).	Youth-friendly health communication and psychosocial support Short courses
**Interpersonal**		
	“We need **safe online spaces for peers to connect** (including crisis support in those spaces). I can also imagine online social gaming spaces used and adapted for positive social and mental health.” (25–35).	Safe, online peer support spaces
**Personal**		
	“Individuals have the power within themselves. Wellness is a purposeful thing done by coercion of the human will in the ever changing environment in order to attain its highest potential. Youths are in that stage where **they need to be equipped with skills** to promote positive mental wellbeing.” (18–24).	Skills building

Sample quotes from *n* = 256 respondents regarding changing ideas about the city and youth mental health during the pandemic. Suggested actions are highlighted in bold.

### Policy and environment

#### Governance and equity

Freedom from discrimination and the value of equity were listed among the mental health-friendly city characteristics; however, respondents pointed out the dearth of equity that COVID-19 unveiled (see the first quote in Table 2).

Respondents observed that policy responses to COVID-19, including mandated curfews and quarantines, shifted the social and economic environment of cities. Young people and their families lost economic opportunities, and cities also became less affordable during the pandemic. Participants explained that poverty and job loss worsened young people’s mental health and well-being and exposed youth to more risk factors because they needed to “hustle or work to place food on the table”. The loss of jobs also deprived youth of hope and underlined the economic inequities that some felt marked their generation more than previous ones. One participant (18–24) reported “Before, I used to think youths need someone who can understand them, empathize with them, but looking at the current scenario, I feel youths need security and a hopeful future too”. In some settings, these economic shifts resulted in an exodus from cities. A respondent (18–24) observed “Cities have always attracted young people but since the pandemic started the cost of living has gone from being a barrier to being another factor in encouraging young people to leave”.

#### Urban built environment

For those who remained in the city, the urban built environment could also offer respite from pandemic-related restrictions in mobility when green spaces and other open spaces were accessible. Participants alluded to cramped urban housing, crowded slums and poor housing infrastructure as stressors that the availability of safe public spaces alleviated. Green space in particular provided solace for young people. A participant (18–24) responded “It’s difficult when you’re confined to the limited space especially when you’re not closer to nature. Negative thoughts get you one way or another even if you try your best. Pandemic has caused more depression I reckon among the youths”. Accessible green space was highlighted as a need and an area for investing effort and policy change (Table 2). A desire for clean, youth-friendly green space for safe gathering and recreation was contrasted with unplanned land use and confined spaces, the latter of which some participants linked to greater risks for young people.

### Community and organizations

Respondents reported diminished access to education and health care, and a disregard of young people’s needs by decision-makers (Table 2). Some responses criticized the lack of forethought before the pandemic to budget for and provide supportive learning environments for youth of all socioeconomic strata. The closure of schools generated stress for young people with the disruption of routines and opportunities to socialize. The pandemic generated greater uncertainty about job opportunities and future trajectories. At the same time, the pandemic brought opportunities to position youth as either contributors and leaders or detractors from community life. Young people reflected on how they experienced inclusion, empathy and exclusion, as well as opportunity for leadership. One respondent (25–35) commented “Our worlds are changing and with it many of our expectations about our education, work, personal interactions and relationships. Instead of being met with understanding, we are collectively positioned as transgressors of social distancing in a way that fails to understand that we are often incredibly vulnerable in this new world and left exposed by lack of infrastructure, service provision and support”.

A respondent (18–24) noticed possibilities for involving young people in responses that could mitigate their numerous losses: “Given the opportunities and resources, young people can be a carrier of change and wellbeing if adults trust them enough to be”.

### Interpersonal domain

Getting through difficult times required interpersonal supports: connectedness through in-person encounters in safe spaces, complemented by digital interactions. Multiple respondents emphasized the relationship between social isolation and poor mental health among city youth during the pandemic, noting the difficulty of making meaningful connection during a time of physical isolation. Two young respondents (18–24) said the well-being of young people was linked to being “in a group of people”, which provides “safety and unity”, and to “inclusion, activity, and interpersonal relationships”. Space repeatedly emerged as a theme, as a conduit to facilitate social connection for young people without risk of COVID-19 transmission, violence, sexual abuse or exposure to drug use. Some participants called for greater investment in creating strong, safe virtual communities for young people; however, although participants identified virtual spaces as a resource for mental health support, a young panellist (18–24) remarked of social media and technology that “It isolated people, even though we have … ways of staying connected 24/7, we still feel lonely.”

### Personal

Consistent with the lead mental health-friendly city characteristic in the personal domain (Figs. 3–5), the pandemic prompted realization of the need for personal skills development to support youth mental well-being. Some respondents expressed concern about the loss of social skills among young people as a result of confinement and an 18–24-year-old commented “… Youths are in that stage where they need to be equipped with skills to promote positive mental wellbeing”. Another young person (18–24) remarked “Most of us do not really have the capacity and necessary skills to support each other when it comes to mental health”. Participants described the importance of being prepared for unpredictable circumstances and enabling youth to “manage themselves, their emotions, and wellbeing”.

### Pandemic-related gains

In some cases, the pandemic brought positive experiences for young people, including more time for self-reflection and discovery, engaging in healing practices, more opportunities to connect with friends, and overall, a greater societal and individual focus on strengthening mental health. A participant (25–35) referred to young people: “They are more conscious about health and their wellbeing by reducing workload and connecting with nature”. Others believed the pandemic revealed young people’s capacity to adapt and to consider the needs of their elders. Some viewed the social justice uprisings that occurred in many countries as a positive vehicle for change and cooperation with others. Changing these conditions would require longer-term solutions: strengthening urban infrastructure and addressing the underlying drivers of inequity. Another participant (>35) lauded the power of youth activism: “… the pandemic has shown us that the resilience of youth is great, as well as the commitment and solidarity with their communities through volunteering, advocacy and youth mobilization”.

## Discussion

Our study convened a multinational and multidisciplinary panel of researchers, practitioners, advocates and young people to identify the characteristics of a mental health-friendly city for youths. The characteristics are distributed among six socioecological domains (Figs. 3–5) that encompass the personal development of young people, supportive educational systems, people-centred health care, a built environment responsive to the needs of young people, and equity-focused policy-making and governance. Within each of these domains, the characteristics we identified are associated with an evolving evidence base linked to youth mental health outcomes and to potential policy intervention.

Intrapersonal characteristics in our list underline the centrality of enabling young people to cultivate skills to manage their interior lives. The targets of such skills-building activities align with proposed ‘active ingredients’ of mental health interventions, such as intervention components related to mechanisms of action or clinical effects on depressive or anxiety symptoms^35^. Examples include affective awareness skills that enable young people to differentiate and describe emotions^36^ and emotion regulation skills to increase and maintain positive emotions^37^. Youth-friendly mental health and educational services, a priority theme at the community level of the framework, could support the intrapersonal realm by deploying a variety of interventions for self-control that benefit adolescent and young adult academic, behavioural and social functioning^38^. Such interventions can also be implemented in earlier childhood educational settings through integration into the curriculum or through other community-based medical or social service organizations^39^. Interventions implemented in selected high-income settings include Promoting Alternative Thinking Strategies^40^, the Incredible Years^41^ and Family Check-up^42^. For young adults, interventions that convey skills to alleviate common psychological problems such as procrastination, perfectionism, low self-esteem, test anxiety and stress could potentially reduce the prevalence of specific mental health conditions while possibly providing acceptable and non-stigmatizing options for care^43,44^.

Our data suggest that a defining theme of any mental health-friendly city for youth is the quality of young people’s social fabric and the city’s ability to provide young people with the skills, opportunities and places required to build and maintain healthy social relationships with their peers, across generations, and as members of a community. The relationships of concern in the interpersonal realm have intrinsic value for healthy adolescent and youth development, promoting well-being^45^ and prevention of depression^46,47^. Panellists also linked opportunities to socialize and build social networks to the availability of safe spaces, the top-ranked priority in the community domain. Achieving safety necessitates equitable and violence-free institutions and cities^48^, a priority that panellists ranked first for ease of implementation in the policy domain. Thus, policies and legislation are required that reduce neglect, bullying, harassment, abuse, censorship, exposure to violence and a wide range of threats towards young people, from homelessness to crime to intimidation by officials^48,49^.

Exposure to community violence and household violence consistently worsens mental health outcomes for youth^50–53^; successful reduction of urban violence should be prioritized. Equity-focused responses to safety needs should include reducing discriminatory physical and structural violence against young people based on race, ethnicity, gender, sexuality or mental health status, which place youth at risk of harmful exposures: rape or trafficking of adolescent girls or police killings of North American Black youth. To create urban spaces in which young people can experience safety, freedom and belongingness requires approaches that actively prevent discrimination^54^ and that consider young people’s multiple identities in the design of institutional as well as outdoor spaces. Women-only parks create greater security for girls and young women and potentially more positive social interaction in some settings^55^.

The benefits of green space, measured as self-satisfaction for adolescents, are linked to greater social contact (for example, more close friends), underscoring space as a conduit for social connection^55^. The advantages of healthy urban spaces for adolescents have emerged not only in health sciences research but also in allied fields such as urban design and sociology^27,56,57^. Urban spaces with opportunities for active commute options to and from school are associated with increased physical activity and environmental supportiveness^58^. Similarly, the presence of community spaces, such as town centres, is associated with improved social connectedness and sense of belonging^59^.

The critical importance of social connectedness was reinforced in the COVID-19 responses. Yet, in many cities the pandemic eliminated spaces that foster urban conviviality, often with lasting effects^60^. Restricted movement and COVID-19 transmission risk associated with public transport may have contributed to greater stress for urban dwellers and ongoing reluctance to use these services^61^. Such factors contribute to social isolation, which may persist in the near term. Consistent with our COVID-19 data, responses from a sample of Australian youth identified social isolation, interrupted education and work, and uncertainty about the future among the primary negative effects of COVID-19 pandemic^62^. In several studies, loneliness increased the risk of mental health conditions among young people during prior epidemics; of relevance to the COVID-19 pandemic, the duration of loneliness predicted future mental health problems^63^.

Analysis of our survey 2 data revealed differences in the priorities of young participants (18–24 and 25–35) compared with panellists over age 35. This discrepancy could have implications for urban decision-makers whose plans to implement positive actions on behalf of young people may not align with what is most salient for youth. Thus, youth involvement in policy development is even more crucial. Soliciting youth perspectives about what supports their mental health based on their personal experiences could simplify and improve interventions intended for them^64^. Several actions could facilitate meaningful youth engagement in governance: encourage collaboration between governments and youth organizations to co-create and co-lead national action plans; implement mechanisms within global governance organizations for youth consultation at local, national and international levels; require inclusion of young people on relevant conference agendas; and improve access to funding for youth-led organizations^65,66^.

Notably, the themes of equity and elimination of discrimination due to race, gender, sexual orientation and neurodiversity arose frequently in the responses to the survey and the COVID-19 question, as did the adversities to which minoritized groups are vulnerable (for example, community violence, police violence and bullying; Figs. 4 and 5). A city that is free of discrimination and racism ranked first among policy responses with immediacy of impact on the mental health of youth—even though no statements proposed dismantling systems of oppression that underlie racism and discrimination, as one respondent noted (Fig. 4). Globally, racism, xenophobia and other forms of discrimination increase mortality and harm the mental health of affected groups through stress-related physiological responses, harmful environmental exposures and limited access to opportunities and health services^20,67–69^. Embedded racist and xenophobic norms, policies and practices of institutions—including those that govern educational, labour and health care systems—yield racialized outcomes for young people around the world (for example, high incidence of HIV infection among adolescent girls in southern sub-Saharan Africa)^20^. To disrupt these forces requires multiple approaches, including recognition and remedy of historical injustices, the activism of social movements committed to change, and implementation of legal frameworks based in human rights norms^70^.

When participants ranked characteristics for ease of implementation (Figs. 3–5), they coalesced around a broad set of factors demonstrating the need for collaboration across urban sectors (for example, normalizing seeking mental health care, promoting democratic cooperation and equal opportunity, and creating employment opportunities and progressive educational systems). This need for cooperation is perhaps most apparent for actions that increase equity. Successful cooperation requires a clear, shared vision and mission, allocation of funding in each sector, diversity of funding sources, distributed decision-making and authority across sectors, and policies that facilitate collaboration^71^. However, well-intentioned cross-sectoral responses to urban needs may inadvertently increase inequities by designing programmes influenced by market forces that magnify environmental privilege (that is, unequal exposure to environmental problems according to social privilege)^54^. Examples include gentrification and development that use land to create green spaces but further dislocate and marginalize communities in need of affordable housing^54^. Implementing community- and youth-partnered processes for urban health equity policy co-creation could yield unified agendas and help to circumvent inequitable outcomes^54,72^. A mental health-friendly city must be positioned to support, integrate and enable the thriving of marginalized and vulnerable young people of the society, who should be involved in its governance.

## Strengths and limitations

Our study has several strengths. First, this priority-setting study yielded a rich dataset of recommended characteristics of a mental health-friendly city for young people from a globally diverse panel of more than 480 individuals from 53 countries. Second, we welcomed expertise from participants with roles relevant to urban sectors: researchers, policymakers and practice-based participants, and we engaged young people in the study advisory board and as study participants, capitalizing on their lived experience. Third, we captured information about how the COVID-19 pandemic influenced participants’ ideas about urban adolescent mental health. Fourth, to our knowledge, this is the first study that brings together a large and multidisciplinary set of stakeholders concerned for cities (for example, urban designers) and for youth mental health (for example, teachers and health professionals) to identify priorities for intersectoral action.

**Extended Data Fig. 1:**
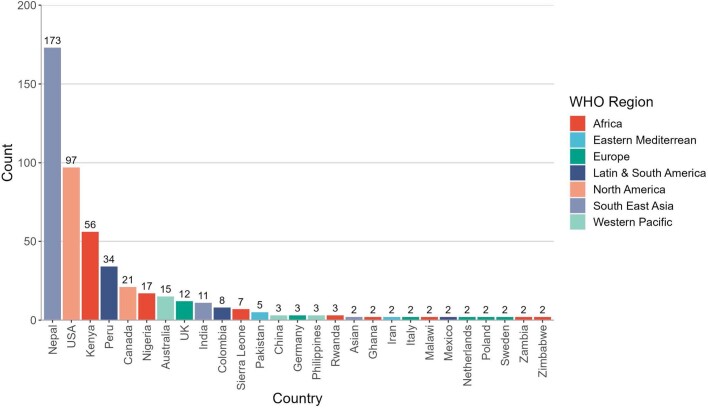
Distribution of Participants by Nationality (N = 518)^a,b,c^. ^a^Countries Participating: Argentina, Australia, Bangladesh, Cameroon, Canada, China, Colombia, Croatia, Czech Republic, Ecuador, Egypt, Ethiopia, France, Germany, Ghana, Haiti, Hong Kong, India, Iran, Italy, Kenya, Malawi, Mauritius, Mexico, Nepal, Netherlands, New Zealand, Nigeria, Norway, Pakistan, Papua New Guinea, Peru, Philippines, Poland, Rwanda, Samoa, Sierra Leone, Slovenia, South Africa, South Korea, Sweden, Switzerland, Taiwan, Tanzania, The Gambia, Tunisia, Turkey, Uganda, UK, USA, Venezuela, Zambia, Zimbabwe (53 total); ^b^Two responses (“Asian” and “Indigenous and European”) do not list a nation but capture verbatim open-text responses; ^c^Countries with one participant removed from graph and include: Argentina, Bangladesh, Cameroon, Croatia, Czech Republic, Ecuador, Egypt, Ethiopia, France, Haiti, Hong Kong, Indigenous and European, Mauritius, New Zealand, Norway, Papua New Guinea, Samoa, Slovenia, South Africa, South Korea, Switzerland, Taiwan, Tanzania, The Gambia, Tunisia, Turkey, Uganda, Venezuela.

**Extended Data Fig. 2:**
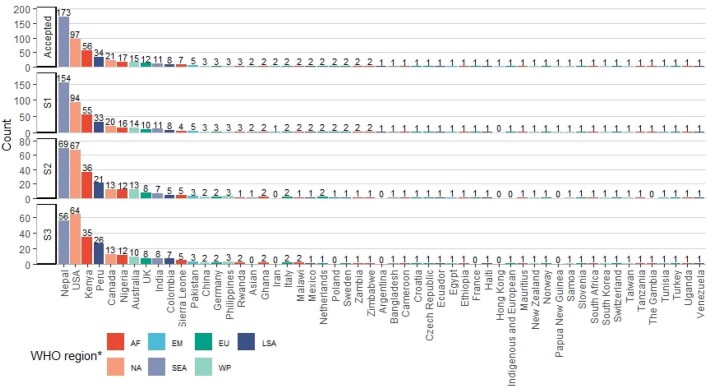
Participant Nationality by Survey Round. ^a^SEA = South-East Asia, NA = North America*, AF = Africa, LSA = Latin & South America*, EU = Europe, WP = Western Pacific, EM = Eastern Mediterranean.

**Extended Data Fig. 3:**
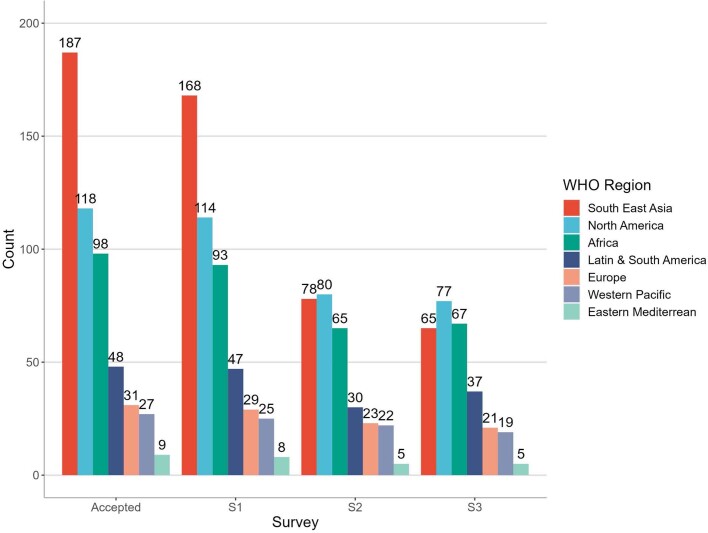
Distribution of Participants by WHO Region^*^ and Survey Round. ^a^SEA = South-East Asia, NA = North America*, AF = Africa, LSA = Latin & South America*, EU = Europe, WP = Western Pacific, EM = Eastern Mediterranean; *We separated North America from Latin & South America for more transparent display of participant distribution.

Our study also has several limitations. First, the participants recruited do not reflect the full social and economic diversity of urban populations whom city governments and decision-makers must serve. Our decision to use a web-based format following standard health research priority-setting methods required tradeoffs. We sought disciplinary, age and geographic diversity; however, our sample does not represent the most marginalized groups of adolescents or adults. Rather, the recruitment of academics, educators, leaders and well-networked young people through an online study probably minimizes the number of participants living in adversity. Although we also recruited young people who were not necessarily established experts, many were students or members of advocacy or international leadership networks and were not likely to exemplify the most disadvantaged groups. We risk masking the specific viewpoints or needs of marginalized and at-risk young people. However, we are reassured by the prominence of equity as a theme and the call to address social determinants of health. Second, it is possible that participants recruited through the authors’ professional networks may be more likely to reflect the viewpoints of the advisory committee members who selected them, given collaborative or other professional relationships. This may have shaped the range of responses and their prioritization. Third, the aspirational calls for an end to discrimination and inequalities highlighted in our results require confronting long-standing structural inequities both within and between countries. Structural violence frequently maintains these power imbalances. Although we do not view their aspirational nature as a limitation, we note that our study data do not outline the complexity of responses required to address these determinants of mental health or to dismantle discriminatory structures. Fourth, our data present several aggregated characteristics that may require disaggregation as cities contextualize the findings for their settings. Fifth, our network recruitment strategy led to skewed recruitment from some geographic regions (for example, North America and Nepal), which may have biased responses (Extended Data Figs. 1–3). Extended Data Table 1 shows the similarities and differences in the rankings for Nepal, USA and the remaining countries in survey 3. Additionally, we recruited few 14–17-year-olds. We experienced attrition over the three rounds of surveying, ending with complete responses from 261 individuals from 48 countries, with the greatest loss in participants between surveys 1 and 2 (Table 1), among the 14–17-, 18–24- and 25–35-year-old age groups, and among participants from Nepal (Extended Data Fig. 2).

## Conclusions

We identified a set of priorities for cities that require intervention at multiple levels and across urban sectors. A clear next step could involve convenings to build national or regional consensus around local priorities and plans to engage stakeholders to co-design implementation of the most salient characteristics of a mental health-friendly city for youth in specific cities (Box 1). It is likely that many variables (for example, geography, politics, culture, race, ethnicity and sexual identity) will shape priorities in each city. Therefore, essential to equitable action is ensuring that an inclusive community of actors is at the table formulating and making decisions, and that pathways for generating knowledge of mental health-friendly city characteristics remain open. This includes representation of sectors beyond mental health that operate at the intersection of areas prioritized by young people. Preparing for implementation will require avenues for youth participation and influence through collective action, social entrepreneurship and representation in national, regional and community decision-making. Enlisting the participation of youth networks that bring young people marginalized owing to sex, gender, sexual orientation, race, economic status, ethnicity or caste; young people with disabilities; and youth and adults with lived experience of mental health conditions in the design of mental health-friendly cities will help to level power imbalances and increase the likelihood that cities meet their needs.

Action for adolescent mental health aligns well with actions nations should take to achieve development targets, and collective action to draw attention to these areas of synergy could benefit youth and cities. Specifically, supporting the mental health of young people aligns with Sustainable Development Goal 11 (sustainable cities and communities) and the New Urban Agenda that aims to “ensure sustainable and inclusive urban economies, to end poverty and to ensure equal rights and opportunities … and integration into the urban space”^73–75^.

Additionally, the list of mental health-friendly city characteristics presents a starting point for strengthening the evidence base on intervening at multiple levels (for example, individual, family, community, organizations and environment) to better understand what works for which youth in which settings. Cities function as complex systems, and systems-centred research can best enable us to understand how individuals’ interactions with one another and with their environments influence good or poor mental health^76^. Similarly, interdisciplinary inquiry is needed that investigates urban precarity and sheds light on social interventions for youth mental health^77^. New research that tests implementation strategies and measures mental health outcomes of coordinated cross-sectoral interventions in cities could be integrated with planned actions. Innovative uses of data that measure the ‘racial opportunity gap’ can help cities to understand how race and place interact to reduce economic well-being for minoritized young people on their trajectory to adulthood^78^. Even heavily studied relationships, such as mental health and green space, can benefit from new methodologies for measuring exposures, including application of mixed methods, and refined characterization of outcomes by gender and age with a focus on adolescents and youth^79^. Globally, mental health-supporting actions for young people in urban areas have an incomplete evidence base, with more peer-reviewed publications skewed towards North American research^73^.

Designing mental health-friendly cities for young people is possible. It requires policy approaches that facilitate systemic, sustained intersectoral commitments at the global as well as local levels^80^. It also requires creative collaboration across multiple sectors because the characteristics identified range from transport to housing to employment to health, with a central focus on social and economic equity. Acting on these characteristics demands coordinated investment, joint planning and decision-making among urban sectoral leaders, and strategic deployment of human and financial resources across local government departments that shape city life and resources^75,81^. This process will be more successful when cities intentionally and accountably implement plans to dismantle structural racism and other forms of discrimination to provide equitable access to economic and educational opportunities for young people, with the goal of eliminating disparate health and social outcomes. The process is made easier when diverse stakeholders identify converging interests and interventions that allow them each to achieve their goals.

Box 1 Considerations for implementing a mental health-friendly city for youth Considerations for implementing a mental health-friendly city for youth using a structure adapted from UNICEF’s strategic framework for the second decade of life^82^ and integrating selected characteristics identified in the study with examples distilled from scientific literature and from project advisory group members. Objectives for implementation along with corresponding examples and selected initiatives are shown.

**Objectives**

- Youth are equipped with resources and skills for personal and emotional development, compassion, self-acceptance, and flourishing.
- Youth develop and sustain safe, healthy relationships and strong intergenerational bonds in age-friendly settings that respect, value and validate them.
- Communities promote youth integration and participation in all areas of community life.
- Communities establish and maintain safe, free public spaces for youth socializing, learning and connection.
- Institutions facilitate satisfying, secure employment; progressive, inclusive, violence-free education; skills for mental health advocacy and peer support.
- Policies support antiracist, gender equitable, non-discriminatory cities that promote democratic cooperation and non-violence.
- Urban environments provide safe, reliable infrastructure for basic amenities and transportation; affordable housing; access to green and blues space; and access to recreation and art.
- Cities minimize adverse social determinants of health; design for safety and security for vulnerable groups; and orient social and built environments to mental health promotion, belonging and purpose.

**Principles**

- Use rights-based approaches
- Prioritize equity for racially, ethnically, gender, sexually and neurologically diverse young people
- Ensure sustained and authentic participation of youth

**Platforms**

- Schools and other educational settings
- Health and social services
- Families and communities
- Religious and spiritual institutions
- Child protection and justice systems
- Peer groups
- Civil society
- Digital and non-digital media

**Implementation objectives**

- Build consensus and contextualize the mental health-friendly city approach at local, regional, national levels
- Engage diverse youth in co-design of mental health-friendly city plans
- Expand opportunities for youth governance
- Enable collaboration among sectors for policy alignment
- Engage communities, schools, health services, media for intervention delivery
- Legislate social protection policies
- Scale interventions to improve economic and behavioral outcomes
- Link implementation to achievement of national or international objectives

**Selected implementation strategies**

- Youth co-design and participation: Growing Up Boulder is an initiative to create more equitable and sustainable communities in which young people participate and influence issues that affect them. It is a partnership between local schools, universities, local government, businesses and local non-profit organizations in the USA that has enabled young people to formally participate in visioning processes such as community assessments, mapping, photo documentation and presentations to city representatives^83^.
- Engaging schools for interventions: universal school-based interventions for mental health promotion^84^; linkage to mental health care for school-based programs^85^; “Whole-school approaches” that engage students and families, communities, and other agencies to support mental health and improve academic outcomes^84,86^.
- Digital platforms for youth mental health: Chile’s HealthyMind Initiative digital platform launched during the COVID-19 pandemic and provided a one-stop resource for information and digital mental health services. The platform included targeted evidence-based resources for children and adolescents^87^.
- Interventions to test at scale: Stepping Stones and Creating Futures is a community-based intervention for intimate partner violence reduction and strengthening livelihoods in urban informal settlements in South Africa that reduced young men’s perpetration of intimate partner violence and increased women’s earning power^88^.
- Shared international objectives: support Sustainable Development Goal 11 and New Urban Agenda targets and Sustainable Development Goals 1–6, 8, 10 and 16.

## Methods

### Project structure and launch

This study aimed to identify priorities for creating cities that promote and sustain adolescent and youth mental health. Central to achieving this aim was our goal of engaging a multidisciplinary, global, age-diverse group of stakeholders. As we began and throughout the study, we were cognizant of the risk of attrition, the importance of maintaining multidisciplinary participation throughout the study and the value of preserving the voices of young people. We used a priority-setting methodology explicitly aimed to be inclusive while simultaneously limiting study attrition. To ensure that we were inclusive of the voices of young people and our large and diverse sample, we limited our study to three surveys, which we determined a priori. Our approach was informed by standard methodologies for health research priority setting^32^.

The project was led by a collaborative team from the University of Washington Consortium for Global Mental Health, Urban@UW, the University of Melbourne and citiesRISE. We assembled three committees representing geographic, national, disciplinary, gender and age diversity to guide the work. First, a core team of P.Y.C., T.W., G.P., M.S. and T.C., generated an initial list of recommended members of the scientific advisory board on the basis of their research and practice activities related to adolescent mental health or the urban setting. We sought a multidisciplinary group representing relevant disciplines. The 18-member scientific advisory board, comprising global leaders in urban design and architecture, social entrepreneurship, education, mental health and adolescent development, provided scientific guidance. We invited members of an executive committee, who represented funding agencies as well as academic and non-governmental organizational leadership, to provide a second level of feedback. A youth advisory board, recruited through citiesRISE youth leaders and other global mental health youth networks, comprised global youth leaders in mental health advocacy. A research team from the University of Washington (Urban@UW, the University of Washington Population Health Initiative and the University of Washington Consortium for Global Mental Health) provided study coordination. The study received institutional review board approval at the University of Washington (STUDY00008502). Invitations to advisory groups were sent in December 2019, along with a concept note describing the aims of the project, and committee memberships were confirmed in January 2020. In February 2020, the committees formulated the question for survey 1: “What are the characteristics of a mental health friendly city for young people?”.

### Study recruitment

The members of the scientific advisory board, youth advisory board and executive committee were invited to nominate individuals with expertise across domains relevant to urban life and adolescent well-being. The group recommended 763 individuals to join the priority-setting panel; individuals invited to serve on the scientific advisory board, youth advisory board and executive committee were included in panel invitations (*n* = 38). Our goal was to establish a geographically diverse panel of participants with scientific, policy and practice-based expertise corresponding to major urban sectors and related challenges (for example, health, education, urban planning and design, youth and criminal justice, housing and homelessness, and violence). Many of the nominees were experts with whom the core group and scientific advisory board members had collaborated, as well as individuals recruited on the basis of their participation in professional and scientific associations and committees (for example, Lancet Commissions and Series) or global practice networks (for example, Teach for All). Nominees’ names, the advisory member who nominated them, gender, country and discipline were tracked by T.C. We used snowball sampling to recruit participants from geographic regions that were under-represented: an additional 24 people were recruited through referrals. The scientific advisory board and youth advisory board sought to maximize the number of young people participating in the study, and invitations were extended to adolescents and young adults through educational, professional, advocacy and advisory networks. Nominees received an invitation letter by e-mail, accompanied by a concept note that introduced the study, defined key constructs, described the roles of the study advisory groups and provided an estimated study timeline. Youth participants (14–24) received a more abbreviated introductory letter. A link to a REDCap survey with an informed consent form and round 1 question was embedded in the invitation e-mail, which was offered in English and Spanish. Of the 824 individuals invited, 518 individuals from 53 countries provided informed consent and agreed to participate, resulting in a nomination acceptance rate of 62.8%.

### Data collection

We administered a series of three sequential surveys using REDCap version 9.8.2. Panellists were asked to respond to the survey 1 question “What are the characteristics of a mental health friendly city for young people?” by providing up to five characteristics and were invited to use as much space as needed. In survey 2, panellists received 134 characteristic statements derived from survey 1 data and were asked to select their 40 most important statements. From these data, we selected 40 most frequently ranked statements. These were presented in the round 3 survey with three redundant statements removed. The remaining 37 characteristic statements were categorized across 6 socioecological domains and panellists were asked to select 1 of 3 framings by which to rank the statements in each domain: immediacy of impact on youth mental health in cities, ability to help youth thrive in cities, and ease or feasibility of implementation. Of individuals who consented to participate, 93.4% completed round 1, 58.5% completed round 2 and 56.2% completed round 3 (Table 1).

We added a new open-ended question to survey 2: “How has the COVID-19 pandemic changed your ideas about the wellbeing of young people in cities?”. Panellists were invited to respond using as many characters (that is, as much space) as needed.

### Data analysis

#### Three-survey series

We managed the survey 1 data using ATLAS.ti 8 software for qualitative data analysis and conducted a conventional content analysis of survey 1 data^89^. Given the multidisciplinarity of the topic and our multidisciplinary group of respondents, we selected an inductive method of analysis to reflect, as simply as possible, the priorities reported by the study sample without imposing disciplinary frameworks. In brief, responses were read multiple times, and characteristics were highlighted in the text. A list of characteristics (words and phrases) was constructed, and we coded the data according to emerging categories (for example, accessibility, basic amenities, career, built environment, mental health services and so on). The analysis yielded 19 broad categories with 423 characteristics. Within each category, characteristics were grouped into statements that preserved meaning while streamlining the list, which yielded 134 characteristic statements. The University of Washington research team convened a 1-week series of data discussions with youth advisers to review the wording of the characteristics and ensure their comprehensibility among readers from different countries. The survey 1 categorized data were reviewed by members of the scientific advisory board, who recommended that using relevant domains to group characteristics would provide meaningful context to the final list. We used IBM SPSS 28.0 for quantitative analyses of data from surveys 2 and 3. In survey 2, we analysed the frequency of endorsement of the 40 characteristics selected by panellists and generated a ranked list of all responses, with the most frequently endorsed at the top. The decision to select 40 characteristics aligned with methods applied in a previous priority-setting exercise^90^ and permitted a list of preferred characteristics that could subsequently be categorized according to a known framework, allowing city stakeholders a broad list from which to select actions. We also analysed frequency of endorsement by age categories (18–24, 25–35 and >35). To amplify the viewpoints of younger participants (under age 35), we combined the top 25 characteristic statements of panellists over 35 with the top 26 characteristic statements of participants under 35 to generate a list of 40 statements, including 11 shared ranked characteristics. As noted, we removed three of these statements because of their redundancy. In survey 3, we analysed data consisting of 37 characteristic statements divided across 6 socioecological domains. Characteristics in each domain were ranked according to one of three framings. We calculated mean ranking and standard deviation for characteristics in each framing category per socioecological domain. Mean rankings (with standard deviation) were calculated across framing categories to arrive at the total mean rank per characteristic and they reflect the proportional contribution of each domain. We also calculated the frequency with which panellists ranked each characteristic statement number 1.

Our study methods align with good practices for health research priority setting as follows^32^.

- Context: we defined a clear focus of the study.
- Use of a comprehensive approach: we outlined methods, time frame and intentions for the results before beginning the study; however, we modified (that is, simplified) the methods for survey 3 to minimize study attrition.
- Inclusiveness: we prioritized recruiting for broad representation and maintaining engagement of an inclusive participant group, and methodological decisions were made in service of this priority.
- Information gathering: our reviews of the literature showed that a study bringing together these key stakeholders had not been conducted, despite the need.
- Planning for implementation: we recognized from the outset that additional convening at regional levels would be required to implement action, and our network members are able to move the agenda forwards.
- Criteria: we determined criteria for the priorities (framing: feasibility of implementation, immediacy of impact and ability to help youth thrive) that study participants used and which we believe will be useful for practical implementation.
- Methods for deciding on priorities: we determined that rank order would be used to determine priorities.
- Evaluation: not applicable; we have not planned an evaluation of the impact of priority setting in this phase of work.
- Transparency: the manuscript preparation, review and revisions enable us to present findings with transparency.

#### COVID-19 qualitative data

We managed the COVID-19 qualitative data using Microsoft Excel and Microsoft Word. We carried out a rapid qualitative analysis^91^. First, the text responses were read and re-read multiple times. We coded the data for content related to expressions of change, no change or areas of emphasis in participants’ perceptions of youth mental health in cities during the pandemic. We focused our attention on data that highlighted changes. We further segmented the data by participant age categories, domains of change and suggested actions, and we assigned socioecological level of changes. We created a matrix using excerpted or highlighted text categorized according to these categories. Three data analysts (P.Y.C., T.C. and A.M.-K.) reviewed the domains of change and identified emerging themes, which were added to the matrix and linked to quotes. The team discussed the themes and came to consensus on assignment to a socioecological level. We prioritized reporting recurring concepts (for example, themes of loss, inequity, green space, isolation and mental illnesses) and contrasting concepts (for example, gains associated with COVID-19) and associated actions^92^.

### Reporting summary

Further information on research design is available in the Nature Portfolio Reporting Summary linked to this article.

## Online content

Any methods, additional references, Nature Portfolio reporting summaries, source data, extended data, supplementary information, acknowledgements, peer review information; details of author contributions and competing interests; and statements of data and code availability are available at 10.1038/s41586-023-07005-4.

### Supplementary information

Supplementary Information Supplementary Note which describes citiesRISE and lists the project team members of Making cities mental health-friendly for adolescents and young adults.

Reporting Summary

## Figures and Tables

**Extended Data Table 1:**
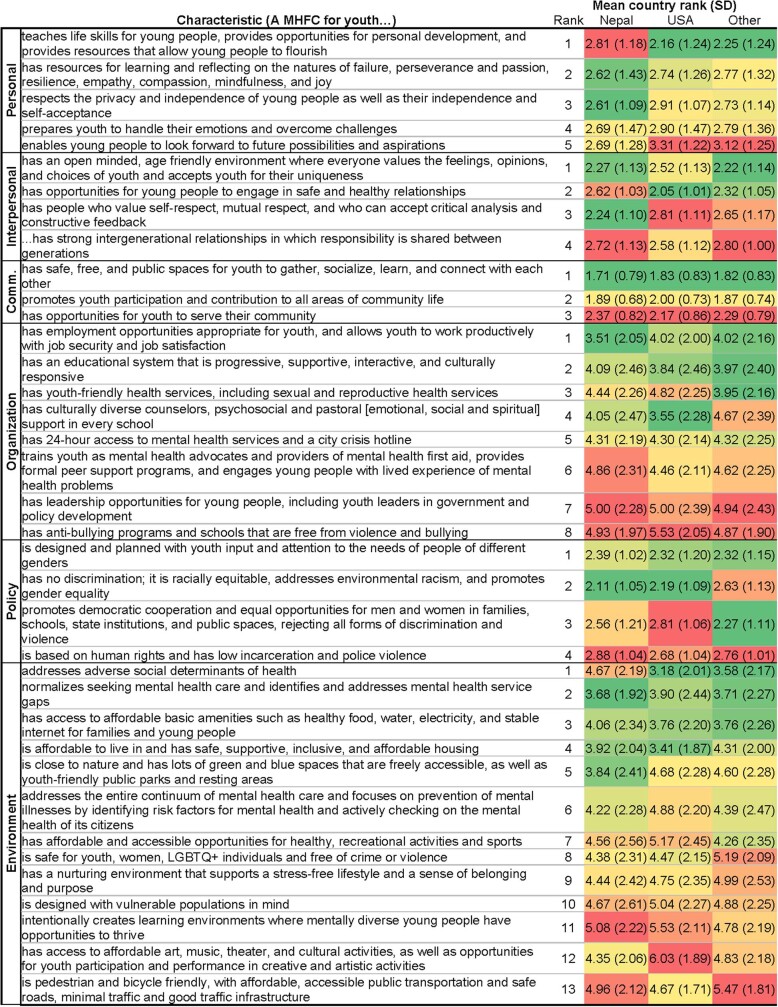
Rankings of MHFC Characteristics by Socio-Ecological Level for USA, Nepal, and other countries

This table shows the total mean rank for each characteristic adjacent to country-specific ranks for 3 categories of data: Nepal, USA, and all other countries. Nepal and the USA represent the sites from which the largest number of participants were recruited. Mean country ranks for each characteristic within a level are shaded from green to red where green represents the highest rank and red represents the lowest rank. Shading highlights where rankings from the largest participant groups diverge or converge relative to each other and to the total mean ranking.

## Data Availability

Survey data that support the findings of this study are available from the corresponding author, P.Y.C., on reasonable request. The sharing of data must comply with institutional policies that require a formal agreement (between the corresponding author and the requester) for sharing and release of data under limits permissible by the institutional review board.

## References

[1] Gruebner O (2017). Cities and mental health. Deutsch. Arztebl. Int..

[2] van der Wal JM (2021). Advancing urban mental health research: from complexity science to actionable targets for intervention. Lancet Psychiatry.

[3] UNICEF Innovation & ARM. *Innovation for Children in an Urbanizing World: a Use-Case Handbook*, https://www.unicef.org/innovation/reports/innovation-children-urbanizing-world (UNICEF, 2017).

[4] Galea S (2011). The urban brain: new directions in research exploring the relation between cities and mood-anxiety disorders. Depress. Anxiety.

[5] March D (2008). Psychosis and place. Epidemiol. Rev..

[6] Faris, R. & Dunham, H. *Mental Disorders in Urban Areas* (Univ. Chicago Press, 1939).

[7] de Leeuw, E. in *Healthy Cities: The Theory, Policy, and Practice of Value-Based Urban Planning* (eds de Leeuw, E. & Simos, J.) Ch. 1, 3–30 (Springer, 2017).

[8] Duhl, L. J. *The Urban Condition: People and Policy in the Metropolis* (Simon and Schuster, 1963).

[9] Vlahov, D., Ettman, C. K. & Galea, S. in *Urban Health* (eds Galea, S. et al.) Ch. 44 (Oxford Univ. Press, 2019).

[10] *Urbanization and Development: Emerging Futures* (UN-Habitat, 2016).

[11] Anglin DM (2021). From womb to neighborhood: a racial analysis of social determinants of psychosis in the United States. Am. J. Psychiatry.

[12] Hancock, T. & Duhl, L. *Promoting Health in the Urban Context* WHO Healthy Cities Papers No. 1 (FADL, 1986).

[13] Okkels N, Kristiansen CB, Munk-Jørgensen P, Sartorius N (2018). Urban mental health: challenges and perspectives. Curr. Opin. Psychiatry.

[14] Kessler R, Berglund P, Demler O, Jin R, Merikangas K (2005). Lifetime prevalence and age-of-onset distributions of DSM-IV disorders in the National Comorbidity Survey replication. Arch. Gen. Psychiatry.

[15] *Global Health Data Exchange, Global Burden of Disease Study 2019* (Institute for Health Metrics and Evaluation, 2020).

[16] Santomauro DF (2021). Global prevalence and burden of depressive and anxiety disorders in 204 countries and territories in 2020 due to the COVID-19 pandemic. Lancet.

[17] Jones S (2022). Mental health, suicidality, and connectedness among high school students during the COVID-19 pandemic - adolescent behaviors and experiences survey, United States, January-June 2021. Morb. Mortal. Wkly Rep. Suppl..

[18] Call K (2002). Adolescent health and well-being in the twenty-first century: a global perspective. J. Res. Adolesc..

[19] Dahl RE, Allen NB, Wilbrecht L, Suleiman AB (2018). Importance of investing in adolescence from a developmental science perspective. Nature.

[20] Selvarajah S (2022). Racism, xenophobia, and discrimination: mapping pathways to health outcomes. Lancet.

[21] Hurd NM, Stoddard SA, Zimmerman MA (2013). Neighborhoods, social support, and African American adolescents’ mental health outcomes: a multilevel path analysis. Child Dev..

[22] *Protecting Youth Mental Health: The U.S. Surgeon General’s Advisory (ed. Health and Human Services)* (Office of the Surgeon General, 2021). 34982518

[23] Patton GC (2016). Our future: a Lancet commission on adolescent health and wellbeing. Lancet.

[24] Bundy DAP (2018). Investment in child and adolescent health and development: key messages from Disease Control Priorities, 3rd Edition. Lancet.

[25] Caruthers AS, Van Ryzin MJ, Dishion TJ (2014). Preventing high-risk sexual behavior in early adulthood with family interventions in adolescence: outcomes and developmental processes. Prev. Sci..

[26] Stelmach R (2022). The global return on investment from preventing and treating adolescent mental disorders and suicide: a modelling study. BMJ Glob. Health.

[27] Roe, J. & McCay L. *Restorative Cities: Urban Design for Mental Health and Wellbeing* (Bloomsbury Visual Arts, 2020).

[28] Knöll M, Roe JJ (2017). Ten questions concerning a new adolescent health urbanism. Build. Environ..

[29] Domaradzka A (2018). Urban social movements and the right to the city: an introduction to the special issue on urban mobilization. Voluntas.

[30] Sinha M, Collins P, Herrman H (2019). Collective action for young people’s mental health: the citiesRISE experience. World Psychiatry.

[31] Sinha M (2020). Towards mental health friendly cities during and after COVID-19. Cities Health.

[32] Viergever RF, Olifson S, Ghaffar A, Terry RF (2010). A checklist for health research priority setting: nine common themes of good practice. Health Res. Policy Syst..

[33] Bronfenbrenner, U. Toward an experimental ecology of human development. *Am. Psychol.* **32**, 513–531 (1977).

[34] Banati, P. & Lansford, J. E. in *Handbook of Adolescent Development Research and its Impact on Global Policy* (eds Lansford, J. E. & Banati, P.) Ch. 1, 1–26 (Oxford Univ. Press, 2017).

[35] *What Science Has Shown Can Help Young People with Anxiety and Depression: Identifying and Reviewing the ‘Active Ingredients’ of Effective Interventions* (Wellcome Trust, 2021).

[36] Beames JR, Kikas K, Werner-Seidler A (2021). Prevention and early intervention of depression in young people: an integrated narrative review of affective awareness and Ecological Momentary Assessment. BMC Psychol..

[37] Daros AR (2021). A meta-analysis of emotional regulation outcomes in psychological interventions for youth with depression and anxiety. Nat. Hum. Behav..

[38] Johnson SB, Voegtline KM, Ialongo N, Hill KG, Musci RJ (2023). Self-control in first grade predicts success in the transition to adulthood. Dev. Psychopathol..

[39] Pandey A (2018). Effectiveness of universal self-regulation-based interventions in children and adolescents: a systematic review and meta-analysis. JAMA Pediatr..

[40] Arda T, Ocak S (2012). Social competence and promoting alternative thinking strategies - PATHS preschool curriculum. Educ. Sci. Theory Pract..

[41] Webster-Stratton C (1984). Randomized trial of two parent-training programs for families with conduct-disordered children. J. Consult. Clin. Psychol..

[42] Hentges RF (2020). The long-term indirect effect of the early Family Check-Up intervention on adolescent internalizing and externalizing symptoms via inhibitory control. Dev. Psychopathol..

[43] Cuijpers P (2021). The associations of common psychological problems with mental disorders among college students. Front. Psychiatry.

[44] Cuijpers P (2021). Indirect prevention and treatment of depression: an emerging paradigm?. Clin. Psychol. Eur..

[45] Blum RW, Lai J, Martinez M, Jessee C (2022). Adolescent connectedness: cornerstone for health and wellbeing. Brit. Med. J..

[46] Filia K, Eastwood O, Herniman S, Badcock P (2021). Facilitating improvements in young people’s social relationships to prevent or treat depression: a review of empirically supported interventions. Transl. Psychiatry.

[47] Herrman H (2022). Time for united action on depression: a Lancet-World Psychiatric Association Commission. Lancet.

[48] United Nations Children’s Fund. *The State of the World’s Children 2021: on My Mind – Promoting, Protecting and Caring for Children’s Mental Health* (UNICEF, 2021).

[49] Massetti, G. M., Hughes, K., Bellis, M. A. & Mercy, J. in *Adverse Childhood Experiences* (eds Asmundson, G. J. G. & Afifi, T. O.) 209–231 (Academic, 2020).

[50] Bordin I (2009). Severe physical punishment: risk of mental health problems for poor urban children in Brazil. Bull. World Health Organ..

[51] Cecil CA, Viding E, Fearon P, Glaser D, McCrory EJ (2017). Disentangling the mental health impact of childhood abuse and neglect. Child Abuse Negl..

[52] Giovanelli A, Reynolds AJ, Mondi CF, Ou SR (2016). Adverse childhood experiences and adult well-being in a low-income, urban cohort. Pediatrics.

[53] Molano A, Harker A, Cristancho JC (2018). Effects of indirect exposure to homicide events on children’s mental health: evidence from urban settings in Colombia. J. Youth Adolesc..

[54] Cole H (2017). Can healthy cities be made really healthy?. Lancet Public Health.

[55] Dadvand P (2019). Use of green spaces, self-satisfaction and social contacts in adolescents: a population-based CASPIAN-V study. Environ. Res..

[56] Markevych I (2014). Access to urban green spaces and behavioural problems in children: results from the GINIplus and LISAplus studies. Environ. Int..

[57] Thompson, C., Silvereirinha de Oliveira, E., Wheeler, B., Depledge, M. & van den Bosch, M. *Urban Green Spaces and Health* (WHO Regional Office for Europe, 2016).

[58] Buli BG, Tillander A, Fell T, Bälter K (2022). Active commuting and healthy behavior among adolescents in neighborhoods with varying socioeconomic status: the NESLA study. Int. J. Environ. Res. Public Health.

[59] Laine J (2014). Cost-effectiveness of population-level physical activity interventions: a systematic review. Am. J. Health Promot..

[60] Martínez L, Short JR (2021). The pandemic city: urban issues in the time of COVID-19. Sustainability.

[61] Mouratidis K (2021). How COVID-19 reshaped quality of life in cities: a synthesis and implications for urban planning. Land Use Policy.

[62] Bell IH (2023). The impact of COVID-19 on youth mental health: a mixed methods survey. Psychiatry Res..

[63] Loades ME (2020). Rapid systematic review: the impact of social isolation and loneliness on the mental health of children and adolescents in the context of COVID-19. J. Am. Acad. Child Adolesc. Psychiatry.

[64] Ng MY, Eckshtain D, Weisz JR (2016). Assessing fit between evidence-based psychotherapies for youth depression and real-life coping in early adolescence. J. Clin. Child Adolesc. Psychol..

[65] O’Kane, C., Haj-Ahmad, J. & Friscia, F. *Engaged and Heard! Guidelines on Adolescent Participation and Civic Engagement*, https://www.unicef.org/media/73296/file/ADAP-Guidelines-for-Participation.pdf (United Nations Children’s Fund, 2020).

[66] Rahmaty, M. & Leiva Roesch, J. *Youth Participation in Global Governance for Sustaining Peace and Climate Action* International Peace Institute Issue Briefs, https://www.ipinst.org/2021/04/youth-participation-in-global-governance-for-sustaining-peace-and-climate-action (International Peace Institute, 2021).

[67] Erondu NA, Mofokeng T, Kavanagh MM, Matache M, Bosha SL (2023). Towards anti-racist policies and strategies to reduce poor health outcomes in racialised communities: introducing the O’Neill-Lancet Commission on Racism, Structural Discrimination, and Global Health. Lancet.

[68] Fani N, Carter SE, Harnett NG, Ressler KJ, Bradley B (2021). Association of racial discrimination with neural response to threat in Black women in the US exposed to trauma. JAMA Psychiatry.

[69] Fani N (2022). Racial discrimination associates with lower cingulate cortex thickness in trauma-exposed black women. Neuropsychopharmacology.

[70] Abubakar I (2022). Confronting the consequences of racism, xenophobia, and discrimination on health and health-care systems. Lancet.

[71] Towe VL (2016). Cross-sector collaborations and partnerships: essential ingredients to help shape health and well-being. Health Aff..

[72] Walker SC (2022). Cocreating evidence-informed health equity policy with community. Health Serv. Res..

[73] Murphy LE, Jack HE, Concepcion TL, Collins PY (2020). Integrating urban adolescent mental health into urban sustainability collective action: an application of Shiffman & Smith’s framework for global health prioritization. Front. Psychiatry.

[74] *New Urban Agenda* (UN Habitat III Secretariat, 2017).

[75] *Health as the Pulse of the New Urban Agenda: United Nations Conference on Housing and Sustainable Urban Development, Quito* (World Health Organization 2016).

[76] Diez Roux AV (2015). Health in cities: is a systems approach needed. Cad. Saude Publica.

[77] Pykett J (2023). Urban precarity and youth mental health: an interpretive scoping review of emerging approaches. Soc. Sci. Med..

[78] O’Brien R, Neman T, Seltzer N, Evans L, Venkataramani A (2020). Structural racism, economic opportunity and racial health disparities: evidence from U.S. counties. SSM Popul. Health.

[79] Fleckney P, Bentley R (2021). The urban public realm and adolescent mental health and wellbeing: a systematic review. Soc. Sci. Medicine.

[80] de Leeuw, E. & Simos, J. Healthy cities move to Maturity in *Healthy Cities* (eds de Leeuw, E. & Simos, J.) Ch. 5, 74-86 (Springer, 2017).

[81] *Our Cities, Our Health, Our Future: Acting on Social Determinants for Health Equity in Urban Settings - Report to the WHO Commission on Social Determinants of Health from the Knowledge Network on Urban Settings* (World Health Organization, 2008).

[82] *UNICEF Programme Guidance for the Second Decade: Programming with and for Adolescents* Programme Division 2018, https://www.unicef.org/media/57336/file (UNICEF, 2018).

[83] *Growing Up Boulder: Boulder’s Child- and Youth-Friendly City Initiative* (Growing Up Boulder, 2015).

[84] O’Reilly M, Svirydzenka N, Adams S, Dogra N (2018). Review of mental health promotion interventions in schools. Soc. Psychiatry Psychiatr. Epidemiol..

[85] Kutcher S (2019). Creating evidence-based youth mental health policy in sub-Saharan Africa: a description of the integrated approach to addressing the issue of youth depression in Malawi and Tanzania. Front. Psychiatry.

[86] Shinde S (2018). Promoting school climate and health outcomes with the SEHER multi-component secondary school intervention in Bihar, India: a cluster-randomised controlled trial. Lancet.

[87] *A New Agenda for Mental Health in the Americas: Report of the Pan American Health Organization High-Level Commission on Mental Health and COVID-1*9, 10.37774/9789275127223 (Pan American Health Organization, 2023).

[88] Gibbs A (2020). Stepping Stones and Creating Futures intervention to prevent intimate partner violence among young people: cluster randomized controlled trial. J. Adolesc. Health.

[89] Hsieh H-F, Shannon SE (2005). Three approaches to qualitative content analysis. Qual. Health Res..

[90] Collins PY (2011). Grand challenges in global mental health. Nature.

[91] Hamilton, A. B. *Qualitative Methods in Rapid Turn-around Health Services Research.* VA HSR&D National Cyberseminar Series: Spotlight on Women’s Health, 2013, https://www.hsrd.research.va.gov/for_researchers/cyber_seminars/archives/video_archive.cfm?SessionID=780 (2013).

[92] Ryan GW, Bernard HR (2003). Techniques to identify themes. Field Methods.

