# Supplementary material for: Making cities mental health friendly for adolescents and young adults
Source: Nature. 2024 Feb 21;627(8002):137–48. doi: 10.1038/s41586-023-07005-4 (PMC10917657; doi:10.1038/s41586-023-07005-4)
Supplement: Supplementary file 1 — Supplementary Note which describes citiesRISE and lists the project team members of Making cities mental health-friendly for adolescents and young adults. [file 41586_2023_7005_MOESM1_ESM.pdf]

nature portfolio

https://doi.org/10.1038/s41586-023-07005-4

Supplementary information

Making cities mental health friendly for adolescents and young adults

In the format provided by the authors and unedited

SUPPLEMENTARY NOTES:

What is citiesRISE?

citiesRISE (https://www.cities-rise.org/) was founded as a multi-stakeholder initiative in 2017 to drive action in the youth mental health field, including paradigm shifts in how mental health is conceptualized and addressed. Over the past five years, citiesRISE has worked collaboratively with young people, communities, professionals, and system leaders across five diverse cities (Bogotá, Colombia; Chennai, India; Nairobi, Kenya; Sacramento and Seattle, U.S.) and beyond to develop innovative evidence-based, scalable ways of supporting significant, durable mental health improvements. At the core of the citiesRISE approach is a commitment to transdisciplinary work, as well as to engaging young people as the center of transformation, working in partnership with other stakeholders. The three pillars of citiesRISE's strategy are:

- Conduct research and innovation projects focused on the core ingredients of effective interventions for youth mental health through a network of city innovation hubs;
- Strengthen and spread the core ingredients via a global Accelerator & Learning Platform supporting social innovators and other stakeholders; and
- Grow a global movement of Mental Health Friendly Cities for Youth (MHFC) driving collective action among young people, system leaders, and innovators in cities around the world.

citiesRISE's approach to working in cities leverages them as places of innovation (city innovation hubs). Through a network of city innovation hubs, citiesRISE brings together young people, communities, professionals, system leaders, and other stakeholders to identify key opportunities and entry points, particularly in the places where young people are (schools, colleges, communities) to make cities more mental health friendly. citiesRISE then facilitates collaborative research and innovative work focused on the core ingredients of effective interventions toward more MHFCs.

Making Cities Mental Health-Friendly for Adolescents and Young Adults: Project Team Members

Scientific Advisory Board:
Lukoye Atwoli, Aga Khan University, Nairobi, Kenya
Nicole Bardikoff, Grand Challenges Canada, Toronto, Ontario, Canada
Inez Bustamante, Universidad Cayetano Heredia, Lima, Peru
Yajun Chen, Sun Yat Sen University, Guangzhou, China
Pamela Y. Collins, University of Washington, Seattle, WA, USA
Tarun Dua, World Health Organization, Geneva, Switzerland
Evelyne de Leeuw, University of New South Wales, Australia
Nathaniel Foote, The TruePoint Center, Boston, MA, USA
Helen Herrman, Orygen and University of Melbourne, Melbourne, Australia
Shisir Khanal, Co-Founder, Teach for Nepal, Kathmandu, Nepal
Manasi Kumar, Institute of Excellence in Health Equity, Department of Population Health, New York University, USA and Department of Psychiatry, University of Nairobi, Nairobi, Kenya
Layla McCay, Centre for Urban Design and Mental Health, London, UK
Bina Lefkowitz, Sacramento County Board of Education and Lefkowitz Consulting, Sacramento, CA, USA
Olayinka Omigbodun, University of Ibadan, Ibadan, Nigeria
George Patton, University of Melbourne, Melbourne, Australia
José Miguel Uribe, Pontificia Universidad Javeriana, Bogotá, Colombia
*Jim Vollendroff, University of Washington, Seattle, WA, USA
Thaisa Way, Dumbarton Oaks, Harvard University, Washington, DC, USA

Youth Advisors and University of Washington Research Staff:
Chantelle Booysen, Good South Social Impact Enterprise, KwaZulu Natal, South Africa
Tessa Concepcion, University of Washington, Seattle, WA, USA
Kelly Davis, Mental Health America, New York, NY, USA
Matthew Hughsam, citiesRISE, New York, NY, USA
Damian Juma, Healthy Brains Global Initiative, Nairobi, Kenya
Augustina Mensa-Kwao, University of Washington, Seattle, WA, USA
Modhurima Moitra, University of Washington, Seattle, WA, USA
Emily Queen, Johns Hopkins University, Baltimore, MD, USA
Yvonne Ochieng, citiesRISE-Nairobi, Nairobi, Kenya
Lian Zeitz, co-founder Climate Mental Health Network, Seattle, WA, USA
*Samuel Talam, Amazing Minds Africa, Nairobi, Kenya

Executive Committee:
Nalini Anand, Fogarty International Center, National Institutes of Health, Bethesda, MD, USA
Peter McDermott, Fajara Associates, London, UK
Moitreyee Sinha, citiesRISE, New York, NY, USA
Jürgen Unützer, University of Washington, Seattle, WA, USA
Miranda Wolpert, Wellcome Trust, London, UK

*Advisory board members who were not co-authors
Pamela Y Collins and Augustina Mensa-Kwao, now at Johns Hopkins Bloomberg School of Public Health, were affiliated with the University of Washington during the active phase of the research.
