# Supplementary material for: Making cities mental health friendly for adolescents and young adults
Source: Nature. 2024 Feb 21;627(8002):137–48. doi: 10.1038/s41586-023-07005-4 (PMC10917657; doi:10.1038/s41586-023-07005-4)
Supplement: Supplementary file 2 — Reporting Summary [file 41586_2023_7005_MOESM2_ESM.pdf]

nature portfolio

Corresponding author(s): Pamela Y. Collins

Last updated by author(s): Oct 10, 2023

Reporting Summary

Nature Portfolio wishes to improve the reproducibility of the work that we publish. This form provides structure for consistency and transparency in reporting. For further information on Nature Portfolio policies, see our Editorial Policies and the Editorial Policy Checklist.

Statistics

For all statistical analyses, confirm that the following items are present in the figure legend, table legend, main text, or Methods section.

n/a	Confirmed	
☒	☐	The exact sample size (*n*) for each experimental group/condition, given as a discrete number and unit of measurement
☐	☒	A statement on whether measurements were taken from distinct samples or whether the same sample was measured repeatedly
☒	☐	The statistical test(s) used AND whether they are one- or two-sided<br>*Only common tests should be described solely by name; describe more complex techniques in the Methods section.*
☒	☐	A description of all covariates tested
☒	☐	A description of any assumptions or corrections, such as tests of normality and adjustment for multiple comparisons
☐	☒	A full description of the statistical parameters including central tendency (e.g. means) or other basic estimates (e.g. regression coefficient) AND variation (e.g. standard deviation) or associated estimates of uncertainty (e.g. confidence intervals)
☒	☐	For null hypothesis testing, the test statistic (e.g. *F*, *t*, *r*) with confidence intervals, effect sizes, degrees of freedom and *P* value noted<br>*Give P values as exact values whenever suitable.*
☒	☐	For Bayesian analysis, information on the choice of priors and Markov chain Monte Carlo settings
☒	☐	For hierarchical and complex designs, identification of the appropriate level for tests and full reporting of outcomes
☒	☐	Estimates of effect sizes (e.g. Cohen's *d*, Pearson's *r*), indicating how they were calculated

Our web collection on statistics for biologists contains articles on many of the points above.

Software and code

Policy information about availability of computer code

Data collection	We utilized Research Electronic Data Capture (REDCap) version 9.8.2, a secure web-based application for managing online surveys, for quantitative and qualitative data collection.
Data analysis	We used IBM SPSS 28.0 for quantitative data analysis and ATLAS.ti 8 for qualitative data analysis.

For manuscripts utilizing custom algorithms or software that are central to the research but not yet described in published literature, software must be made available to editors and reviewers. We strongly encourage code deposition in a community repository (e.g. GitHub). See the Nature Portfolio guidelines for submitting code & software for further information.

Data

Policy information about availability of data

All manuscripts must include a data availability statement. This statement should provide the following information, where applicable:

- Accession codes, unique identifiers, or web links for publicly available datasets
- A description of any restrictions on data availability
- For clinical datasets or third party data, please ensure that the statement adheres to our policy

Survey data that support the findings of this study are available from the corresponding author, PYC, on reasonable request. This means that the sharing of data must comply with institutional policies that require a formal agreement (between the corresponding author and the requestor) for sharing and release of data under limits permissible by the Institutional Review Board.

Field-specific reporting

Please select the one below that is the best fit for your research. If you are not sure, read the appropriate sections before making your selection.

☐ Life sciences ☒ Behavioural & social sciences ☐ Ecological, evolutionary & environmental sciences

For a reference copy of the document with all sections, see nature.com/documents/nr-reporting-summary-flat.pdf

Behavioural & social sciences study design

All studies must disclose on these points even when the disclosure is negative.

Study description	The study is a sequential, multi-survey priority-setting exercise comprising two open-ended survey questions that provided qualitative data and 2 structured surveys that permitted quantification of the results.
Research sample	The study sample comprises children (age 14-17 yrs) and adults (18 yrs and older) representing 53 countries.
Sampling strategy	We recruited individuals with practice-based, policy, research expertise and/or lived experience as a young person under the age of 35 through nominations submitted by the project's Scientific Advisory Board. To improve geographic diversity, we used snowball sampling to ensure participation from specific regions. Because the aim of the study was to achieve input and determine priorities from diverse stakeholders, we did not predetermine sample size.
Data collection	Participants were contacted vis email, provided details about the study, and invited to give informed consent for participation in a 3-round survey via REDCap. Participants responded to open-ended questions in the first and second surveys, and they responded to structured survey questions in the second and third surveys using REDCap.
Timing	Data collection began in April 2020 and ended in December 2020. The first survey period occurred from April-May 2020, the second from August to September 2020, and the third from November - December 2020.
Data exclusions	No data were excluded.
Non-participation	825 participants were invited to participate, 307 did not respond. 518 people accepted the invitation, 484 submitted Survey 1 data, 303 submitted Survey 2 data (215, no response), 291 submitted Survey 3 data (227, no response). We did not collect data on reasons for non-participation or drop-out.
Randomization	The study did not involve randomization.

Reporting for specific materials, systems and methods

We require information from authors about some types of materials, experimental systems and methods used in many studies. Here, indicate whether each material, system or method listed is relevant to your study. If you are not sure if a list item applies to your research, read the appropriate section before selecting a response.

Materials & experimental systems

n/a	Involved in the study
☒	☐ Antibodies
☒	☐ Eukaryotic cell lines
☒	☐ Palaeontology and archaeology
☒	☐ Animals and other organisms
☒	☐ Human research participants
☒	☐ Clinical data
☒	☐ Dual use research of concern

Methods

n/a	Involved in the study
☒	☐ ChIP-seq
☒	☐ Flow cytometry
☒	☐ MRI-based neuroimaging
